# Pediatric body composition based on automatic segmentation of computed tomography scans: a pilot study

**DOI:** 10.1007/s00247-023-05739-x

**Published:** 2023-08-29

**Authors:** Atia Samim, Suzanne Spijkers, Pim Moeskops, Annemieke S. Littooij, Pim A. de Jong, Wouter B. Veldhuis, Bob D. de Vos, Hanneke M. van Santen, Rutger A. J. Nievelstein

**Affiliations:** 1 Department of Radiology and Nuclear Medicine, University Medical Center Utrecht and Wilhelmina Children’s Hospital, Heidelberglaan 100, 3584 CX, Utrecht, The Netherlands; 2 Princess Máxima Center for Pediatric Oncology, Utrecht, The Netherlands; 3 Quantib-U, Utrecht, The Netherlands; 4 Image Sciences Institute, University Medical Center Utrecht and Utrecht University, Utrecht, The Netherlands; 5 Department of Biomedical Engineering and Physics, Amsterdam University Medical Centers – location AMC, Amsterdam, The Netherlands; 6 Department of Pediatric Endocrinology, Wilhelmina Children’s Hospital, University Medical Center Utrecht, Utrecht, The Netherlands

**Keywords:** Body composition, Child, Computed tomography, Sarcopenia, Skeletal muscle, Subcutaneous fat, Visceral fat

## Abstract

**Background:**

Body composition during childhood may predispose to negative health outcomes later in life. Automatic segmentation may assist in quantifying pediatric body composition in children.

**Objective:**

To evaluate automatic segmentation for body composition on pediatric computed tomography (CT) scans and to provide normative data on muscle and fat areas throughout childhood using automatic segmentation.

**Materials and methods:**

In this pilot study, 537 children (ages 1–17 years) who underwent abdominal CT after high-energy trauma at a Dutch tertiary center (2002–2019) were retrospectively identified. Of these, the CT images of 493 children (66% boys) were used to establish normative data. Muscle (psoas, paraspinal and abdominal wall) and fat (subcutaneous and visceral) areas were measured at the third lumbar vertebral (L3) level by automatic segmentation. A representative subset of 52 scans was also manually segmented to evaluate the performance of automatic segmentation.

**Results:**

For manually-segmented versus automatically-segmented areas (52 scans), mean Dice coefficients were high for muscle (0.87–0.90) and subcutaneous fat (0.88), but lower for visceral fat (0.60). In the control group, muscle area was comparable for both sexes until the age of 13 years, whereafter, boys developed relatively more muscle. From a young age, boys were more prone to visceral fat storage than girls. Overall, boys had significantly higher visceral-to-subcutaneous fat ratios (median 1.1 vs. 0.6, *P*<0.01) and girls higher fat-to-muscle ratios (median 1.0 vs. 0.7, *P*<0.01).

**Conclusion:**

Automatic segmentation of L3-level muscle and fat areas allows for accurate quantification of pediatric body composition. Using automatic segmentation, the development in muscle and fat distribution during childhood (in otherwise healthy) Dutch children was demonstrated.

**Supplementary information:**

Supplementary material is available at 10.1007/s00247-023-05739-x.

## Introduction

Body composition, in particular the distribution of skeletal muscle and fat tissue, can be an important predictor of various health outcomes [1]. In adults, for instance, sarcopenia has been associated with increased mortality and morbidity [2]; and excess visceral fat deposition has been associated with a higher risk of metabolic syndrome and malignancy [3, 4]. Nevertheless, studies on pediatric body composition and its prognostic significance are limited.

Childhood sarcopenia is a novel concept that needs to be defined. Yet, low muscle mass and loss of muscle are highly prevalent in clinical settings (20–41% of children) [5–10] and are associated with increased morbidity [5, 8–14]. Importantly, the presence of sarcopenia is often overlooked when obtaining anthropometric measurements, particularly when excess body fat is present. In an era of increasing childhood obesity, sarcopenic obesity may be an underrecognized condition.

Currently, cross-sectional imaging (computed tomography [CT] and magnetic resonance imaging [MRI]) is considered the gold standard for body composition analysis. It provides direct anatomical measurements, allowing for the discrimination of separate skeletal muscle and fat (subcutaneous, visceral) distribution [15, 16]. Axial single-slice measurements of abdominal muscle and fat area have shown to reliably predict whole-body muscle and fat mass, respectively [17]. Automatic segmentation tools are increasingly utilized, which are less time-consuming than manual segmentation and less affected by intra- and interobserver variability [18–20].

From birth to adolescence, pediatric body composition changes rapidly due to physiological changes in longitudinal height, endocrine status and energy expenditure. Before these changing body composition measures can begin to have a meaningful prognostic impact, data on the normal variation in cross-sectional body composition are needed. There is a lack of data on values for total muscle and fat areas on cross-sectional imaging in European children.

This study aimed to evaluate an automatic tool for the segmentation of skeletal muscle and fat areas on pediatric CT scans and to provide data on the variation in body composition throughout childhood in a cohort of otherwise healthy Dutch children using automatic segmentation.

## Materials and methods

### Study population

All children, ages 1 to 17 years, who underwent an abdominal CT scan after high-energy trauma (high-impact falls and traffic accidents) at the emergency department of a single Dutch tertiary center, between August 2002 and April 2019, were identified as representative of the general pediatric population (*n*=537). No individual was scanned twice. Patients were excluded in case of evident anatomic anomalies or extensive post-traumatic changes that would prevent reliable segmentation. Clinical data of the patients were not available.

### CT image acquisition

All patients received 2 ml/kg contrast medium lopromide (Ultravist 300, Bayer Healthcare, Berlin, Germany) according to our trauma protocol. CT examinations were obtained with multidetector scanners: Mx8000 IDT 16, Brilliance 64, iQon Spectral or Brilliance iCT; (all Philips Medical Systems, Cleveland, OH). Exposure settings (range: 35–190 mAs and 80–120 kVp) were adjusted to patient size. Axial 4 mm slices were reconstructed and displayed with a standard abdominal soft tissue setting (window level: 30, window width: 400).

### Automatic segmentation tool

The Quantib Body Composition tool was used for automatic segmentation [21, 22] (available online for scans of adults, https://research.quantib.com). As the method was developed for adult CTs, for this study, the networks of the slice selection and segmentation methods were retrained using a set of 49 manually-segmented images in children.

The segmentation method consisted of two steps. First, the automatic tool identified the slice at the craniocaudal midportion of the third lumbar vertebra (L3) from the CT data set using a convolutional neural network. Second, the automatic tool segmented the L3 slice into the following areas, using a second dilated convolutional neural network [23]: psoas, abdominal wall (rectus abdominis; transversus abdominis; internal and external obliques) and paraspinal (quadratus lumborum, erector spinae, multifidus, latissimus dorsi) muscles and subcutaneous and visceral fat. The third lumbar vertebra is the most commonly used level for body composition assessment in the literature [16, 24].

To minimize the influence of the exact slice that was selected, the automatic tool segmented a total of five slices around the detected L3 level, that is, two above and two below the center-L3 slice, and, therefore, averaged the area measurements over a range of 2 cm.

### Manual segmentation

A total of 52 axial CT images (not used during training of the segmentation tool), selected from all age groups and sexes, were manually segmented by one trained observer (A.S., a final-year medical student with 3 months of experience in radiology) using Medical Imaging Interaction Toolkit (MITK) version 2018.04 (www.mitk.org) [25]. In case of uncertainty, a radiologist (P.A.d.J.) with 15 years of experience was consulted. To compute intra-observer agreement, 30 of the 52 scans were segmented twice by the same observer, at least 12 months later, in a different order and blinded to previous results.

### Evaluation of automatic segmentations

Manually-segmented CT images were used as the validation subset for automatic segmentation. Measurements of automatic and manual segmented areas were compared using the intraclass correlation coefficient (ICC) and Bland-Altman plots. To assess correct anatomical decision-making by the algorithm, Dice coefficients were computed between manual and automatic segmentations at the same slice, as well as between the intra-observer segmentations.

### Normative data from automatic segmentations

To obtain normative data, CT scans of all 537 patients were processed with the automatic segmentation tool. Independent evaluation of all automatic segmentations was performed by another reviewer (S.S., with three years of experience in pediatric radiology research). The reviewer excluded all visually incorrect automatic segmentations, in terms of level and/or segmented areas and accepted only small segmentation errors, that would not result in important changes in area measurements. Descriptive statistics were used to report data in the form of median values and interquartile ranges (IQR). The analyses were stratified by sex and age. For each patient, the following ratios were calculated: visceral-to-subcutaneous fat, visceral-to-total fat and total muscle-to-fat area.

Correlations between continuous variables were calculated using Spearman’s rho correlation coefficients. Student’s *T*-tests were used to test the difference between means of continuous variables between the two sexes. Logarithmic transformations were applied to non-normally distributed variables. The data were analyzed using software (SPSS version 25, IBM, Armonk, NY and R Core Team 2017, R Foundation for Statistical Computing, Vienna, Austria). The quantreg package in R was used for the reconstruction of the quantile regression curves (R. Koenker [2020], *https://cran.r-project.org/package=quantreg*). *P-*values <0.05 were considered statistically significant.

## Results

### Automated and manual segmentation

**Fig. 1:**
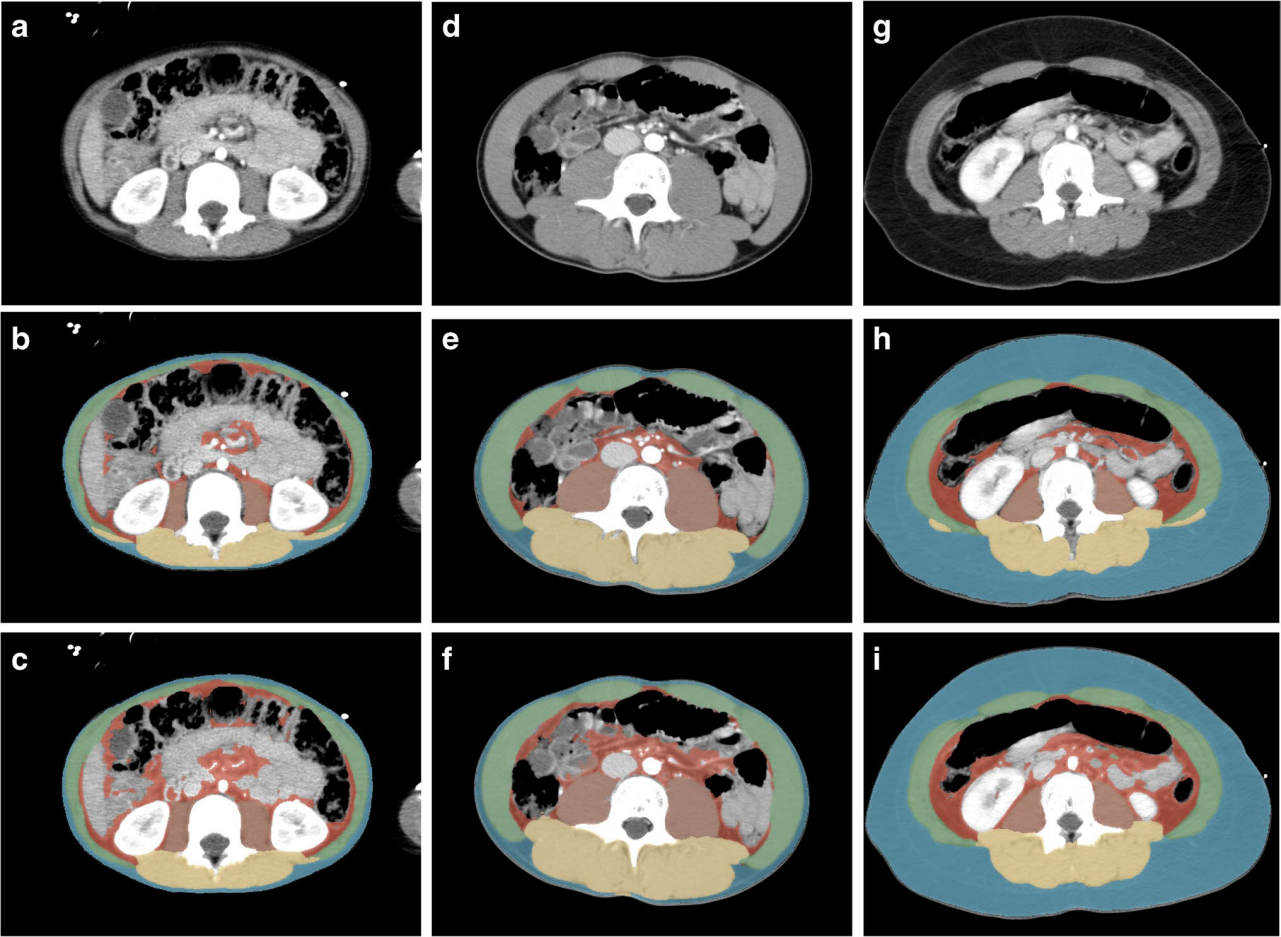
Axial computed tomography images obtained in a 5-year-old boy (**a**–**c**), a 15-year-old boy (**d**–**f**) and a 12-year-old girl (**g**–**i**) at the level of the third lumbar vertebral body performed following high-energy trauma. Contrast-enhanced images before segmentation (**a**, **d**, **g**) and after manual (**b**, **e**, **h**) and automatic (**c**, **f**, **i**) segmentation. Automatic segmentation sometimes included parts of the intestines as visceral fat and often failed to differentiate the abdominal wall muscles from the latissimus dorsi muscles. *Blue* subcutaneous fat, *green* abdominal wall muscles, *red* visceral fat, *orange* psoas muscles, *yellow* paraspinal muscles

**Fig. 2:**
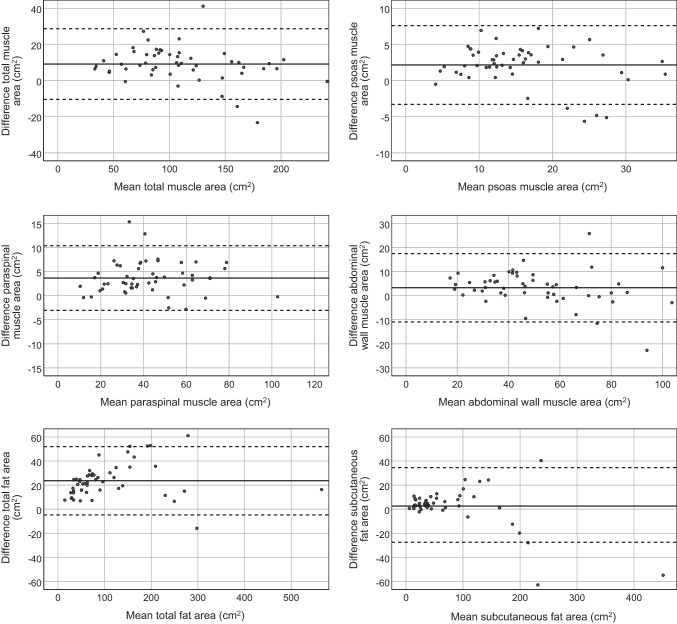
Bland Altman plots comparing measurements by automatic and manual segmentation of muscle and fat areas in 52 children. The difference (automatic - manual) between the two measurements (y-axis) is plotted against their average (x-axis). The broken lines represent the 95% confidence intervals of the average differences and the continuous line represents the mean difference

For the 52 CT examinations used to validate the tool, the ICC between manually- and automatically-segmented areas was 0.98 (95% confidence interval [CI] 0.96–0.99) for total muscle area and 0.99 (95% CI 0.98–0.99) for total fat area (*P*<0.001) (Fig. 1). Bland Altman plots (Fig. 2) show that automatically-segmented measurements of visceral fat and total fat area are systematically higher than the manually-segmented measurements. Dice coefficients for manual vs. automatic segmentation were similar to the Dice coefficients of manual intra-observer variation (Table 1), with the highest variation for visceral fat.

**Table 1:** Dice coefficients for manual and automatic segmentation at the level of the third lumbar vertebral body

	Mean Dice coefficients ± standard deviation	
	Manual vs. manual segmentations (intra-observer)	Manual vs. automatic segmentation
Psoas muscles	0.90 ± 0.03	0.87 ± 0.06
Paraspinal muscles	0.91 ± 0.03	0.90 ± 0.04
Abdominal wall muscles	0.89 ± 0.03	0.87 ± 0.05
Subcutaneous fat	0.86 ± 0.10	0.88 ± 0.09
Visceral fat	0.67 ± 0.09	0.60 ± 0.09

### Normative data

After automatic segmentation of the CT scans of all 537 patients, we excluded CT examinations of 44 patients for the following reasons: 17 because of abnormal anatomy (consisting of lumbosacral agenesis and muscular dystrophia) and/or extensive posttraumatic changes (consisting of burst fracture of L3, emphysema/hemorrhage of intraperitoneal space, visceral organs, muscles and/or subcutis) and 27 because of incorrect CT segmentations in terms of selected level or segmented areas. This resulted in a study sample of 493 children, ages 1 to 17 years, including 326 boys (66%) for the establishment of normative data. Age distribution was similar for both sexes, with a median age of 14 (IQR 9–17) years for boys and 14 (IQR 9–16) for girls.

### Muscle and fat areas at the level of the third lumbar vertebra

**Fig. 3:**
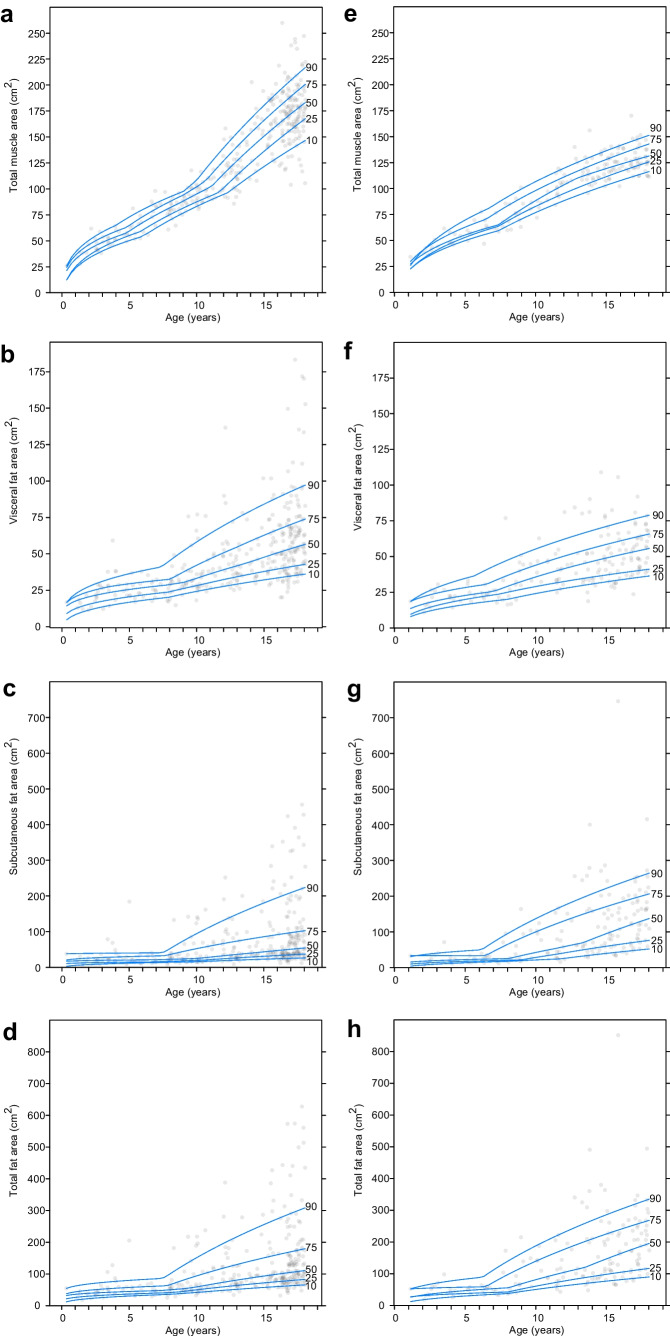
Quantile regression curves at the level of the third lumbar vertebra in boys (**a**–**d**) and girls (**e**–**h**) show total muscle (**a**, **e**) and visceral (**b**, **f**) subcutaneous (**c**, **g**) and total fat (**d**, **h**) areas according to age and sex. Observed 10th, 25th, 50th, 75th and 90th percentiles

Values (median and IQR, Tables 2 and 3) according to age and sex are summarized for L3 total muscle areas (Table 2) and visceral, subcutaneous and total fat area (Table 3). Corresponding quantile regression curves are plotted in Fig. 3 (curves for psoas, abdominal and long spine muscles in Supplementary Material 1, Fig. 1).

**Table 2:** Muscle areas (psoas, paraspinal, abdominal wall and total) according to age and sex at the level of the third lumbar vertebra *IQR* interquartile range

	Boys (*n*=326)					Girls (*n*=167)				
Age, y	*n (%)*	Median (IQR) muscle area, cm^2^				*n (%)*	Median (IQR) muscle area, cm^2^			
		Psoas	Paraspinal	Abdominal wall	Total		Psoas	Paraspinal	Abdominal wall	Total
1	7 (2%)	5 (5–6)	11 (10–14)	19 (15–21)	38 (31–39)	1 (1%)	2	10	22	34
2	9 (3%)	7 (6–8)	17 (15–20)	20 (20–24)	43 (41–47)	7 (4%)	7 (6–7)	14 (13–17)	19 (19–21)	41 (38-43)
3	11 (3%)	7 (6–8)	20 (19–22)	24 (22–25)	51 (48–53)	5 (3%)	8 (8–11)	18 (17–21)	23 (23–26)	48 (47-58)
4	6 (2%)	8 (8–9)	23 (22–23)	26 (25–27)	57 (56–59)	4 (2%)	8 (8–9)	18 (17–20)	23 (22–25)	51 (47-55)
5	10 (3%)	9 (8–10)	23 (21–24)	31 (26–34)	64 (55–68)	5 (3%)	9 (7–10)	21 (30–21)	28 (26–29)	57 (57-60)
6	9 (3%)	12 (9–13)	32 (27–34)	36 (28–39)	78 (65–85)	7 (4%)	9 (8–12)	25 (24–26)	28 (28–30)	62 (61-67)
7	6 (2%)	12 (11–14)	33 (32–34)	35 (32–37)	81 (77–85)	8 (5%)	10 (9–11)	25 (25–28)	29 (27–31)	63 (61-70)
8	18 (6%)	12 (12–14)	33 (30–35)	36 (34–39)	81 (77–87)	10 (6%)	11 (11–12)	28 (26–35)	34 (32–38)	76 (69-83)
9	17 (5%)	14 (13–15)	36 (35–40)	45 (42–46)	93 (88–101)	3 (2%)	12 (11–12)	34 (34–35)	38 (36–40)	85 (83-86)
10	18 (6%)	14 (13–16)	38 (35–41)	42 (39–45)	95 (87–101)	6 (4%)	13 (11–14)	34 (33–37)	38 (36–43)	87 (83-89)
11	11 (3%)	16 (14–17)	40 (39–43)	44 (41–58)	101 (98–114)	12 (7%)	16 (14–17)	41 (38–47)	45 (41–49)	103 (95-108)
12	25 (8%)	17 (14–19)	45 (40–53)	53 (46–57)	115 (102–125)	6 (4%)	18 (16–21)	47 (43–50)	54 (51–57)	119 (109-128)
13	13 (4%)	21 (19–23)	50 (46–58)	55 (49–62)	122 (116–141)	15 (9%)	19 (16–20)	46 (43–49)	52 (48–56)	114 (109-123)
14	14 (4%)	25 (22–27)	64 (49–71)	69 (62–79)	158 (133–169)	15 (9%)	19 (17–21)	46 (44–51)	48 (47–56)	114 (109-128)
15	26 (8%)	26 (23–30)	67 (58–73)	71 (68–79)	165 (149–185)	20 (12%)	18 (17–21)	51 (46–53)	54 (52–58)	125 (117-129)
16	57 (18%)	28 (22–31)	69 (63–76)	72 (68–81)	172 (156–188)	14 (8%)	18 (17–21)	51 (49–56)	55 (53–59)	123 (120-136)
17	69 (21%)	29 (24–31)	72 (65–79)	74 (68–83)	174 (160–190)	29 (17%)	20 (18–21)	52 (48–57)	56 (52–59)	127 (123-138)

**Table 3:** Fat areas (subcutaneous, visceral and total) according to age and sex at the level of the third lumbar vertebra *IQR* interquartile range

	Boys (*n*=326)				Girls (*n*=167)			
Age, y	*n (%)*	Median (IQR) fat areas, cm^2^			*n (%)*	Median (IQR) fat areas, cm^2^		
		Subcutaneous	Visceral	Total		Subcutaneous	Visceral	Total
1	7 (2%)	18 (15–28)	16 (13–18)	37 (31–37)	1 (1%)	34	19	53
2	9 (3%)	20 (10–22)	22 (20–27)	38 (34–46)	7 (4%)	19 (17–30)	17 (15–24)	35 (32–55)
3	11 (3%)	23 (19–37)	27 (20–33	50 (39–72)	5 (3%)	29 (20–41)	23 (22–27)	51 (41–71)
4	6 (2%)	27 (16–30)	27 (25–29)	54 (38–59)	4 (2%)	16 (14–18)	19 (18–20)	35 (32–38)
5	10 (3%)	18 (15–21)	23 (21–29)	45 (36–56)	5 (3%)	21 (15–28)	25 (23–31)	51 (48–52)
6	9 (3%)	18 (15–32)	25 (21–30)	46 (39–58)	7 (4%)	28 (26–33)	24 (23–25)	56 (49–59)
7	6 (2%)	15 (14–17)	25 (19–29)	41 (37–44)	8 (7%)	21 (19–34)	24 (19–28)	44 (37–62)
8	18 (6%)	24 (23–34)	30 (25–33)	55 (49–67)	10 (6%)	25 (20–35)	31 (28–44)	66 (49–87)
9	17 (5%)	22 (17–65)	32 (29–39)	54 (48–100)	3 (2%)	94 (60–99)	44 (39–45)	139 (99–144)
10	18 (6%)	24 (19–52)	35 (28–38)	59 (47–95)	6 (4%)	86 (52–101)	47 (41–47)	124 (94–142)
11	11 (3%)	34 (22–65)	40 (36–49)	83 (59–118)	12 (7%)	51 (27–85)	34 (31–41)	83 (64–124)
12	25 (8%)	45 (27–94)	41 (33–53)	84 (66–150)	6 (4%)	98 (63–146)	49 (44–74)	146 (108–190)
13	13 (4%)	31 (27–58)	42 (36–52)	73 (63–101)	15 (9%)	66 (47–205)	41 (34–52)	112 (79–260)
14	14 (4%)	38 (34–57)	44 (36–56)	91 (71–95)	15 (9%)	68 (52–133)	46 (37–57)	107 (87–196)
15	26 (8%)	45 (36–100)	50 (41–65)	95 (82–164)	20 (12%)	95 (74–148)	45 (32–58)	139 (109–222)
16	57 (18%)	40 (32–93)	50 (37–67)	88 (72–157)	14 (8%)	95 (55–147)	49 (39–55)	142 (93–203)
17	69 (21%)	69 (38–110)	62 (47–76)	129 (90–183)	29 (17%)	165 (105–218)	58 (46–69)	227 (148–274)

With increasing age, median total muscle area ranged from 38 cm^2^ (IQR 31–39) to 174 cm^2^ (IQR 160–190) in boys and from 41 cm^2^ (IQR 38–43) to 127 cm^2^ (IQR 123–138) in girls. A strong correlation between total muscle area and age was found for both sexes (Spearman’s correlation coefficient 0.88 for boys and 0.85 for girls, both *P*<0.01). Furthermore, psoas muscle area was strongly correlated with total muscle area for both sexes (0.94 for boys, 0.88 for girls, both *P*<0.01).

With increasing age, median total fat area increased from 37 cm^2^ (IQR 31–37) to 129 cm^2^ (IQR 90–183) in boys and from 35 cm^2^ (IQR 32–55) to 227 cm^2^ (IQR 148–274) in girls. Total fat areas increased with age for both sexes (with a correlation coefficient of 0.61 for boys and 0.70 for girls, *P*<0.001), especially during adolescence. A proportion (10%) of boys and girls begin to accumulate more fat tissue even before the average age for onset of puberty.

## Ratios

The ratio between visceral and subcutaneous fat decreased with age for both sexes and ranged between 0.1 and 3.5 for boys (median 1.1, IQR 0.7–1.5) and between 0.2 and 3.4 for girls (median 0.6, IQR 0.4–0.9). When comparing the ratio of visceral-to-subcutaneous fat between sexes, a mean difference of 0.4 (*P*<0.01) in visceral-to-subcutaneous fat ratio was observed, indicating a higher percentage of visceral fat in boys than in girls throughout childhood. The total fat-to-muscle ratio ranged from 0.2 to 3.8 in boys (median 0.7, IQR 0.5–1.0) and from 0.4 to 3.9 in girls (median 1.0, IQR 0.8–1.7). The mean difference in total fat-to-muscle ratio between boys and girls was 0.4 (*P*<0.01) with a higher mean ratio in girls. With increasing age, a subtle downward trend in total fat-to-muscle ratio was seen in boys, but a portion of boys exceeded values of 1.0 (Supplementary Material 2, Fig. 2).

## Discussion

In this pilot study, an automatic segmentation tool for body composition was evaluated and used to provide data for total muscle and fat areas at the level of L3 in children undergoing CT after high-energy trauma. Mean Dice coefficients between manually- and automatically-segmented areas were high for muscle (0.87–0.90) and subcutaneous fat (0.88), but lower for visceral fat (0.60). As expected, around puberty boys start to gain more muscle mass compared to girls and the overall fat-to-muscle ratio is higher in girls compared to boys (median 1.01 vs. 0.65; *P*<0.01). Approximately 10% of boys and girls begin to accumulate more fat tissue before the average age for onset of puberty. Our study demonstrates that even from a young age, boys appear more prone to visceral fat storage.

The performance of the automatic tool is generally good when compared with the intra-observer manual segmentations. The low intra-observer Dice coefficients for visceral fat (for both manual and automatic segmentation) demonstrate that visceral fat is the most difficult compartment to segment in children who have little visceral fat. The Bland-Altman analysis revealed systematically higher values of visceral fat area by automatic segmentation compared to manual segmentation. This may be for various reasons: (1) the automatic tool is more sensitive in segmenting small areas and therefore includes more small regions of visceral fat (see example in Fig. 1); (2) the automatic tool used the average of multi-slice segmentation vs. one-slice manual segmentation. Multi-slice assessment may provide a more consistent representation of fat area, compared to one-slice measurement, especially for visceral fat area; (3) the automatic tool overestimates visceral fat at the cost of visceral organs (see example in Fig. 1). All in all, with 5% of the studies being excluded due to incorrect automatic segmentation, automatic segmentation should not be blindly applied without expert oversight and review.

Our L3 values were overall similar to age- and sex-specific measurements of psoas muscle areas at intervertebral spaces L3/L4 and L4/L5 [26] and L4 [27] in a cohort representing children in North America (undergoing CT after trauma and/or appendicitis). Harbaugh et al. [27] also provided data for visceral fat area at L4 in children in the USA. Our IQR for visceral fat were higher than their data, which may be explained by the difference in level and overestimation of visceral fat compared to manual segmentation in our cohort. Studies on multilevel measurements and comparisons between levels in children are limited. Further research comparing different levels in pediatric populations could be valuable for exploring potential variations and establishing standardized protocols.

We have provided insight into the timing of changes in body muscle and fat throughout childhood. While boys and girls have comparable fat and muscle area during early childhood, a wider variance is observed during adolescence. We quantify that with the start of puberty, triggered by the gonadal hormones, boys generally gain more muscle and girls gain more fat. While the amount of visceral fat at birth is known to be negligible [28], we found that children start to accumulate visceral fat from a young age, even before the age of onset of puberty. This increase in fat accumulation may be partially explained by the physiological preparation for puberty. It is well-established that adult males have higher visceral-to-subcutaneous fat ratios and adult females have more subcutaneous fat and total fat [29]. In our cohort, these sex differences in fat distribution were present from a young age. This has also been described by several studies in children of various ages between four and eighteen years [30–33]. The timing of fat accumulation has important implications for future health, including the risk of developing metabolic complications associated with excess adiposity [34].

Body composition analysis has broad relevance in primary and specialty healthcare settings. Normal values are important to identify children with abnormal body composition and to evaluate its prognostic impact. Altered body composition can affect the distribution and clearance of drugs [35, 36]. Children with aberrant body composition may be at risk for under- and overdosing. It should be studied whether personalized body composition-based dosing can minimize treatment toxicity and improve treatment efficacy [37]. In addition, whether improving body composition with supportive (nutritional and physical) interventions can improve outcomes, such as chemotherapy tolerance and quality of life, has yet to be studied in children.

The evaluation of an automatic segmentation tool provides a basis for further validation of body composition as a predictor of outcomes in many disease settings. It is important to establish reference and cut-off values for morphometric measures that can predict an increased risk of adverse outcomes. Cross-sectional imaging is often available in children in the clinical setting and holds important body compositional information. Cross-sectional imaging from, for example, trauma patients can serve as a representation of the general pediatric population. We should not scan children solely for the purpose of pediatric body composition analysis. We advocate for opportunistic use of these readily-available cross-sectional images for routine body composition assessment.

Some limitations of our study should be addressed. First, when interpreting the Dice coefficients, ICC and Bland Altman plots, it should be noted that only 52 scans were segmented manually and scans with visual abnormalities were not included. Second, for some age groups, the sample size per sex was not sufficient to determine reliable normative values, as older children (age 15–17 years) and boys (66%) were overrepresented in our study cohort. Third, some important parameters of body composition were not available, such as a history of chronic disease, pubertal stage or race/ethnicity. It would have been very informative to correlate CT measures to body mass index, but these data were not available because the height/weight of these children is not routinely measured in the trauma setting. Fourth, in this study, we included trauma patients from one single tertiary center in a relatively urban area as the best available representation of the general pediatric population. It could be that children that sustain high-energy trauma differ in body composition from the average child. The automatic tool was trained on a relatively small dataset from a single center, which may limit generalizability. Training the automatic tool using more manually-segmented scans from a more diverse dataset, including a wider range of factors such as age, anatomy, and scan acquisition parameters, could result in a more robust algorithm.

This pilot study demonstrates the pediatric application of an automatic tool for generating data for cross-sectional body composition. In a cohort of the Dutch pediatric population, we found important differences in both L3 muscle and fat areas according to sex and age. Knowledge of these values adds to our understanding of the physiologic changes in body composition that occur throughout childhood. It provides a basis for further studies on (early) identification of vulnerable children with aberrant body composition and evaluation of its possible health risks throughout life.

## Conclusion

With the increasing use of cross-sectional imaging and the development of automated segmentation methods, it is only now that we can harvest the wealth of prognostic power that lies in body-compositional analyses. A logical next step is to not only analyze tissue volumes but include their make-up in terms of CT Hounsfield units, which will further increase the relevance and applicability of these measures. 

### Supplementary information

ESM 1 (DOCX 2.23 MB)

## Data Availability

Source data discussed in the article are available on e-mail request to the guarantors of this study.
