# Supplementary material for: Pediatric body composition based on automatic segmentation of computed tomography scans: a pilot study
Source: Pediatr Radiol. 2023 Aug 29;53(12):2492–501. doi: 10.1007/s00247-023-05739-x (PMC10635977; doi:10.1007/s00247-023-05739-x)
Supplement: Supplementary file 1 — (DOCX 2.23 MB) [file 247_2023_5739_MOESM1_ESM.docx]

**Supplementary Material 1:** Quantile regression curves at the level of the third lumbar vertebra

|  | Boys (*n*=326) | Girls (*n*=167) |
| --- | --- | --- |
| a |  |  |
| b |  |  |
| c |  |  |

Area of the psoas (**a**), abdominal wall (**b**) and paraspinal (**c**) muscles according to age and sex. Observed 10th, 25th, 50th, 75th and 90th percentiles

**Supplementary Material 2:** Scatter plots with trend lines at the level of the third lumbar vertebra

|  | Boys (*n*=326) | Girls (*n*=167) |
| --- | --- | --- |
| a |  |  |
| b |  |  |

Ratios of visceral-to-subcutaneous fat (**a**) and total fat-to-muscle (**b**) per age (years) according to sex
